# Dehydrated Human Amnion/Chorion Grafts May Accelerate the Healing of Ulcers on Free Flaps in Patients With Venous Insufficiency and/or Lymphedema

**Published:** 2016-09-07

**Authors:** Edward P. Miranda, Alex Friedman

**Affiliations:** ^a^Center for Complex Reconstruction, San Francisco, Calif; ^b^Department of Plastic Surgery, California Pacific Medical Center, San Francisco

**Keywords:** amnion/chorion, free flap, microsurgery, venous ulcer, lymphedema

## Abstract

**Objective:** Ulceration of free flaps in patients with venous insufficiency and/or lymphedema is an uncommon but challenging problem. We hypothesized that dehydrated human amnion/chorion membrane (Epifix) grafts would accelerate healing of these challenging ulcers. **Methods:** Retrospective analysis of prospectively acquired data identified 8 lower extremity free flaps with ulcerations in the context of venous insufficiency and/or lymphedema. The first 4 were flaps that had been treated with conservative wound care to healing. The second group was treated conservatively initially but then converted to treatment with dehydrated human amnion/chorion membrane grafts. The primary endpoint was time to healing. **Results:** Comparison of Kaplan-Meier survival curves revealed a significant difference between the conservatively and dehydrated human amnion/chorion membrane–treated flap ulcers, favoring graft treatment (*P* = .0361). In those ulcers that healed, the average time to healing was 87 days for the conservative treatment group and 33 days for the dehydrated human amnion/chorion membrane treatment group (with an average of 1.7 grafts per ulcer). **Conclusions:** Dehydrated human amnion/chorion membrane may accelerate healing of ulcers on lower extremity free flaps in patient with lymphedema and/or venous disease in the treated leg.

Free tissue transfer in patients with venous insufficiency or lymphedema is challenging. Ulcerations of the free tissue flap in these patients are common and often lead to challenging wound-healing problems and prolonged care in and out of the hospital. While successful free tissue transfer has excellent wound-healing outcomes in general, the presence of venous and/or lymphatic congestion directly impairs the final healing of the wound.

Dehydrated amnion/chorion has been shown to accelerate wound healing in lower extremity wounds with venous insufficiency.^1^ Dehydrated human amnion/chorion membrane (dHACM) contains numerous growth factors, tissue inhibitors of matrix metalloproteinases (TIMPs), and cytokines. Growth factors include platelet-derived growth factor-AA (PDGF-AA), PDGF-BB, transforming growth factor α (TGFα), TGFβ1, basic fibroblast growth factor (bFGF), epidermal growth factor (EGF), placental growth factor (PLGF), and granulocyte colony-stimulating factor (GCSF). Interleukins (ILs) and TIMPs have been detected (IL-4, 6, 8, and 10, and TIMP 1, 2, and 4).^2^ Each of these factors influence and may accelerate wound healing.

Free flaps can be performed in the context of venous insufficiency and have been reported to be performed for the treatment of the sequelae of venous insufficiency.^3^ However, wound-healing complication rates have been reported as high as 35% to 43%.^4^^,^^5^ We hypothesized that dHACM grafts would be successful in rapidly resolving ulcers on lower extremity free flaps in patients with documented venous insufficiency and/or lymphedema.

## METHODS

Eight consecutive lower extremity free flaps during a 6-month period were identified in a tertiary care hospital setting that developed postoperative ulceration in the context of confirmed venous insufficiency and/or lymphedema. Each of the 8 ulcerations were treated conservatively (dressing changes and/or compression). The initial 4 cases were treated until healing. The second 4 cases were initially treated conservatively (dressing changes and/or compression) until it was established that the wounds would not heal and then each of this second group was treated with dHACM grafting (Epifix; MiMedx Group, Inc, Marietta, Ga). dHACM grafts were applied at least 1-week intervals, until healing was observed. Multilayer venous compression dressings (Profore; Smith & Nephew, Hull, United Kingdom) were placed over grafts that were below the knee.

The treatments were evaluated for the presence of ulcer healing, number of graft applications, time to healing, and recurrence. Data were collected via retrospective review of prospectively collected data. Data were analyzed by descriptive statistics between the 2 groups. The 2 groups were further evaluated by the Kaplan-Meier survival analysis, with the primary endpoint being healing of the ulcer using Mathematica computational software (Wolfram Research, Champaign, Ill). Censoring was performed for conversion, from conservative wound care to dHACM treatment or for loss of follow-up. Hazard ratios were computed from the survival curves and compared with the log-rank test. A *P* value of less than .05 was considered significant.

## RESULTS

### Participants

Eight free-flap cases were identified, and all 8 were included in the study. One free-flap case in the dHACM group was censored upon conversion from conservative care to dHACM due to repeated intentional removal of the dHACM grafts by the patient. Follow-ups were performed at weekly to monthly intervals as deemed clinically relevant. The mean follow-up was 351 days (range, 58–1006 days). Participant characteristics and comorbidities are reported in Table 1.

### Wound characteristics

Significant edema was present in all treated lower extremities. All flaps had documented venous insufficiency and/or lymphedema. Five flaps (63%) were in the setting of documented lymphedema. Six flaps (75%) were in the setting of venous insufficiency (reflux and/or obstruction). All wounds were superficial ulcers. There were no incidences of flap necrosis or anastomotic revision. The average size of the ulcers was 5.5 cm^2^ (range, 1-15 cm^2^). Wound and flap characteristics are presented in Table 2.

### Conservative therapy and ulcer healing

In the first 4 flaps, dHACM was not available at our institution. Consequently, conservative wound care and graduated compression were employed. The average wound size was 5.0 cm^2^ (range, 2–10 cm^2^). For these flaps, the mean time to healing was 87 days (range, 41–195 days).

### dHACM grafts and ulcer healing

In the dHACM-treated group, an average of 1.7 dHACM grafts were done per flap. Three ulcerated flaps were included in the dHACM treatment arm, and all of these ulcers healed after dHACM grafting. The average wound size and the number of grafts in the subgroup that healed were 6.1 cm^2^ (range, 1–15 cm^2^) and 1.7 (range, 1–2), respectively. The mean time to healing was 33 days (range, 10–66 days).

### Kaplan-Meier analysis

**Figure 1:**
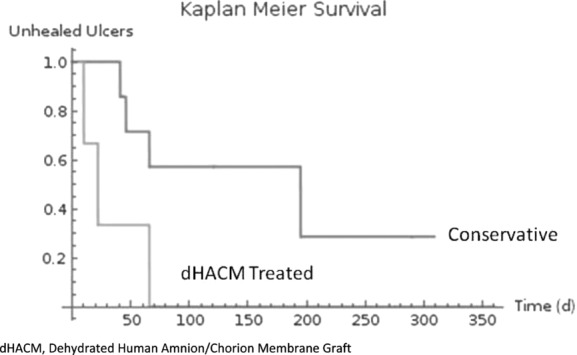
*Ulcer healing over time.* dHACM indicates dehydrated human amnion/chorion membrane.

Kaplan-Meier survival curves for the conservatively treated and dHACM treatment groups are presented in Figure 1. The hazard rates were different based on the log-rank test, with *z* = 2.1 and *P* = .0361, indicating that treatment with dHACM resulted in flap ulcers healing faster than conservative care.

## DISCUSSION

Lower extremity free flaps performed in the context of lymphedema and/or venous insufficiency are prone to subacute superficial ulcerations that are unrelated to anastomotic failure. Typically, we have treated these wounds with prolonged local wound care. Given that dHACM grafts have been shown to be effective in healing venous stasis ulcers, we hypothesized that dHACM would be effective on similar free flap ulcerations. In our report, dHACM ulcers healed faster than conservatively treated free flap ulcers, requiring only 1 or 2 dHACM grafts.

Our study is an initial observational report and has limitations in general applicability. The sample size is small, limiting sweeping conclusions. There is no true randomized control or comparison group available, so it cannot be firmly concluded (based on our results) that dHACM accelerates healing of ulcers on free flaps with lymphedematous or venous-insufficient limbs.

The flap ulcer that was excluded from the dHACM treatment group deserves analysis. This flap and its associated ulcer had several differences from the others. Its location was more proximal than the others (knee vs leg and ankle). The ulcer was present for many months prior to dHACM grafting. Unlike the other treated flaps, dHACM grafts to this flap did not receive compression therapy. Last (and perhaps most importantly) the patient repeatedly excoriated the flap, removing the grafts. dHACM grafting was terminated after 4 grafts.

In conclusion, we demonstrated that dHACM grafting on free flap venous/lymphedema ulcers is technically feasible and may be useful for initiating or accelerating healing. Potential predictors for unsuccessful treatment may include the absence of compression therapy, poor patient compliance, and prolonged time to treatment of the wound. Further study may be required, including a randomized prospective trial.

## Figures and Tables

**Table 1:** Patient characteristics

	All patients	dHACM subgroup
Age, mean (SD), y	63.1 (15.7)	61.7 (13.7)
Diabetes, %	12.50	33.30
BMI, mean (SD), kg/m^2^	34.7 (15.8)	29.0 (3.9)
PAD, %	0.0	0.0
Venous insufficiency, %	75.0	66.7
Lymphedema, %	50.0	66.7

dHACM indicates dehydrated human amnion/chorion membrane; BMI, body mass index; and PAD, peripheral arterial disease.

**Table 2:** Wound characteristics and healing

Obstructive diagnosis	Flap	Location	Indication	Veins	Treatment time, d	Ulcer size, cm^2^	Healing time, d	Follow-up, d	dHACM applications
A. Conservative treatment									
VI	ALT	Leg	Osteomyelitis	2	66	2	66	815	
LE & VI	ALT	Foot	Osteomyelitis	2	46	6	46	1006	
VI	ALT	Leg	Osteomyelitis	1	195	10	195	447	
VI & LE	RFA	Foot	Osteomyelitis	*1*	41	2	41	74	
LE	ALT	Knee	Osteomyelitis	1	309	6.3	*Censored*	89	
VI	Gracilis + STSG	Ankle	Crush and open fracture	1	21	15	Converted to dHACM	253	
LE & VI	ALT	Leg	Crush and degloving	1	90	1	Converted to dHACM	66	
LE	Gracilis + STSG	Ankle	Osteomyelitis	1	80	2	Converted to dHACM	58	
				Mean	106	5.5	87	351	
				SD	97.6	4.9	72.8	373.3	
B. dHACM subgroup									
VI	Gracilis + STSG	Ankle	Crush and open fracture	1	10	15	10	253	1
LE & VI	ALT	Leg	Crush and degloving	1	66	1	66	66	2
LE	Gracilis + STSG	Ankle	Osteomyelitis	1	22	2	22	58	2
				Mean	32.7	6	32.7	125.7	1.7
				SD	29.5	7.8	29.5	110.3	0.6

VI indicates venous insufficiency; LE, lymphedema; ALT, anterolateral thigh perforator; RFA, radial forearm; STSG, split-thickness skin graft; and dHACM, dehydrated human amnion/chorion membrane.
